# Effects of *Perovskia abrotanoides* Kar. Essential Oil on Greenhouse Gas Mitigation and Energy Efficiency Improvement in Sheep Rumen: An In Vitro Study

**DOI:** 10.1002/vms3.71194

**Published:** 2026-08-23

**Authors:** Mohsen Kazemi

**Affiliations:** ^1^ Department of Animal Science Faculty of Agriculture and Animal Science University of Torbat‐e Jam Torbat‐e Jam Iran

**Keywords:** essential oil, in vitro, methane mitigation, *Perovskia abrotanoides*, rumen fermentation

## Abstract

**Background:**

The use of natural additives in animal nutrition, particularly for ruminants, has gained attention due to environmental concerns and the need to improve rumen fermentation efficiency. Essential oils are bioactive sources that can modulate rumen microbes and reduce methane production, a major greenhouse gas and source of energy loss.

**Objectives:**

This study aimed to characterize the chemical composition of *Perovskia abrotanoides* Kar. essential oil and evaluate its potential as a natural rumen modifier by assessing its effects on in vitro rumen fermentation parameters, gas and methane production, protozoa population, nutrient degradability and microbial efficiency.

**Methods:**

Essential oil was extracted by water distillation and analysed using GC/MS. Rumen fluid was collected from three fistulated sheep that fed a maintenance diet. Six levels of essential oil (0, 100, 200, 300, 400 and 500 mg/L culture medium) were evaluated in a completely randomized design. Gas production was measured at 0–96 h, whereas methane, volatile fatty acids (VFAs), ammonia nitrogen (NH_3_–N), pH, protozoa count, degradability and microbial parameters were measured after 24 h. Data were analysed using ANOVA followed by Tukey's multiple comparison test.

**Results:**

GC/MS analysis revealed 33 compounds, with 1,8‐cineole (16.1%), camphor (13.8%), α‐pinene (8.4%) and δ‐3‐carene (6.8%) as the predominant constituents. Increasing concentrations of *P. abrotanoides* essential oil produced dose‐dependent changes in rumen fermentation. The highest supplementation level (500 mg/L) led to the most significant reductions in methane production (58%), total protozoal population, total VFA concentration, NH_3_–N concentration and the degradability of dry matter, organic matter and neutral detergent fibre, while simultaneously producing the highest ruminal pH, microbial biomass production, microbial synthesis efficiency and partitioning factor (*p* < 0.0001). In contrast, supplementation at 200–300 mg/L maintained a more favourable balance between methane mitigation and nutrient degradability.

**Conclusions:**

The findings demonstrate that *P. abrotanoides* essential oil, rich in oxygenated monoterpenes, such as 1,8‐cineole and camphor, effectively reduced methane production and protozoa population while improving microbial efficiency under in vitro conditions. The greatest reduction in methane production and in protozoal population was observed at 500 mg/L, whereas supplementation at 200–300 mg/L provided the best balance between methane mitigation and maintenance of rumen fermentation efficiency. These results suggest that this essential oil could serve as a promising natural feed additive for mitigating enteric methane emissions and improving energy utilization in ruminants. Further in vivo studies are recommended to confirm these effects under practical feeding conditions.

## Graphical abstract

The essential oil of *Perovskia abrotanoides* Kar. (rich in 1,8‐cineole and camphor) linearly reduced methane production (up to 57.5%) and total protozoa population in sheep rumen fluid in vitro. Simultaneously, it increased microbial biomass production, microbial synthesis efficiency and the partitioning factor, indicating improved rumen energy utilization. A moderate dose (200–300 mg/L) offers an optimal balance between methane mitigation and nutrient degradability.

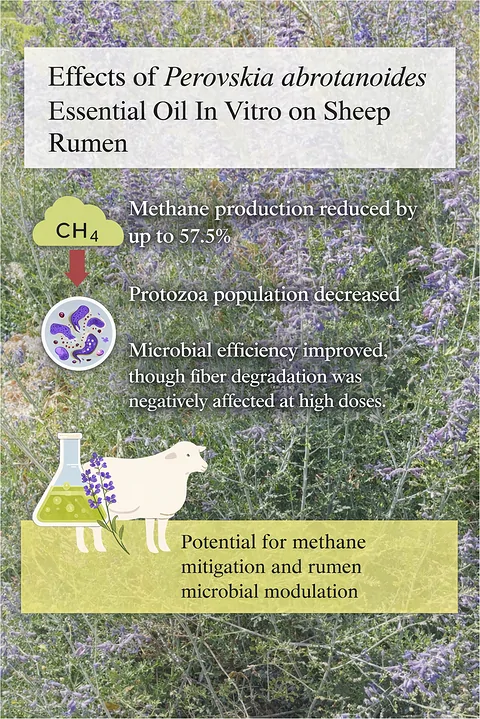

## 1 Introduction

Ruminant livestock production systems face significant challenges, including reduced rumen fermentation efficiency, dietary energy loss as methane and environmental consequences of greenhouse gas emissions. Enteric methane represents a 2%–12% loss of metabolizable energy (ME) from feed and constitutes a major greenhouse gas with substantial global warming potential (Xie et al. 2025). The extensive use of chemical and antibiotic feed additives to improve rumen fermentation efficiency has encountered increasing limitations due to microbial resistance development and potential hazards to human and animal health (Salman et al. 2026). Consequently, attention towards natural additives, particularly plant‐derived compounds, has intensified as sustainable and safe alternatives for modulating rumen fermentation and mitigating methane production (Kazemi 2019; Kazemi and Eskandari Torbaghan 2019). Therefore, identifying natural feed additives capable of simultaneously improving rumen fermentation efficiency and reducing enteric methane emissions has become a major research priority for sustainable ruminant production systems.

Essential oils, as bioactive secondary metabolites of plants, have garnered considerable interest in small ruminant nutrition due to their capacity to modulate rumen fermentation, influence microbial populations and exert antioxidant and anti‐inflammatory effects (Idowu et al. 2025; Nasir et al. 2026). Evidence suggests that essential oil supplementation can simultaneously improve energy and nutrient utilization efficiency while effectively reducing greenhouse gas emissions, particularly methane (Caroprese et al. 2023). Recent studies have further demonstrated that essential oils can selectively suppress methanogenic archaea and ruminal protozoa while modulating bacterial populations involved in fibre degradation (Dorantes‐Iturbide et al. 2022; Muslykhah et al. 2026). However, the efficacy of essential oils varies considerably depending on their botanical origin, chemical composition, dosage and dietary conditions, emphasizing the importance of evaluating individual plant species before their practical application (Yohannis et al. 2026).

*Perovskia abrotanoides* Kar. (Lamiaceae) is a perennial plant widely distributed across arid and semi‐arid regions of Iran and Central Asia (Sajjadi et al. 2005). This species has traditionally been valued in Iranian medicine for its anti‐inflammatory, antiseptic and antimicrobial properties. Phytochemical investigations have demonstrated that *P. abrotanoides* represents a rich source of bioactive compounds, including monoterpenes and sesquiterpenes in the essential oil, alongside diverse phenolic and flavonoid constituents in various extracts (Sajjadi et al. 2005). The essential oil has been reported to contain 1,8‐cineole, camphor, α‐pinene and β‐pinene as major active components responsible for its antibacterial and antifungal activities (Mahboubi and Kazempour 2009; Ashraf et al. 2014). Furthermore, methanolic and ethanolic extracts from different plant organs, particularly flowers, exhibit high total phenolic content and notable antioxidant capacity, indicating substantial potential for inhibiting oxidative processes and microbial activities (Mazandarani et al. 2010; Ashraf et al. 2014). Despite these well‐documented pharmacological and antimicrobial properties, the potential application of *P. abrotanoides* essential oil as a natural rumen modifier has received little scientific attention, and its effects on rumen fermentation characteristics and methane mitigation remain largely unexplored.

Although an increasing number of studies have investigated plant‐derived essential oils as natural rumen modifiers (Nhara and Baloyi 2025; Nasir et al. 2026), the reported effects on fermentation, methane mitigation and nutrient utilization remain inconsistent across literature. Most previous studies have focused on commercial essential oils or a limited set of conventional medicinal plants (Nasir et al. 2025; Muslykhah et al. 2026). Information regarding the biological activity of *P. abrotanoides* essential oil in the rumen ecosystem is still scarce, and the relationship between its unique chemical composition (particularly its high concentration of oxygenated monoterpenes) and its influence on methane production, microbial populations, nutrient degradability and microbial efficiency has not been comprehensively evaluated under controlled in vitro conditions.

Key fermentation parameters, such as gas and methane production, volatile fatty acid (VFA) concentrations, NH_3_–N, protozoal populations and microbial biomass synthesis efficiency, serve as critical indicators for evaluating the effects of feed additives on rumen health and functionality (Patra and Saxena 2009). Comprehensive evaluation of these parameters provides valuable insights into the mechanisms through which bioactive plant compounds influence microbial metabolism, fermentation efficiency and energy utilization in the rumen.

Accordingly, the objectives of the present study were to determine the chemical composition of *P. abrotanoides* essential oil and to investigate the effects of different inclusion levels of whole‐plant essential oil on gas production kinetics, methane production, fermentation parameters, nutrient degradability, protozoal population and microbial efficiency indices in sheep rumen fluid under controlled in vitro conditions. We hypothesized that supplementation with *P. abrotanoides* essential oil would produce dose‐dependent modifications in rumen fermentation, resulting in reduced methane production and protozoal populations while improving microbial biomass synthesis and energy utilization efficiency. Furthermore, we hypothesized that an optimal supplementation level would mitigate methane emissions without adversely affecting overall nutrient degradability.

## 2 Materials and Methods

### 2.1 Plant Materials and Essential Oil Extraction

**FIGURE 1:**
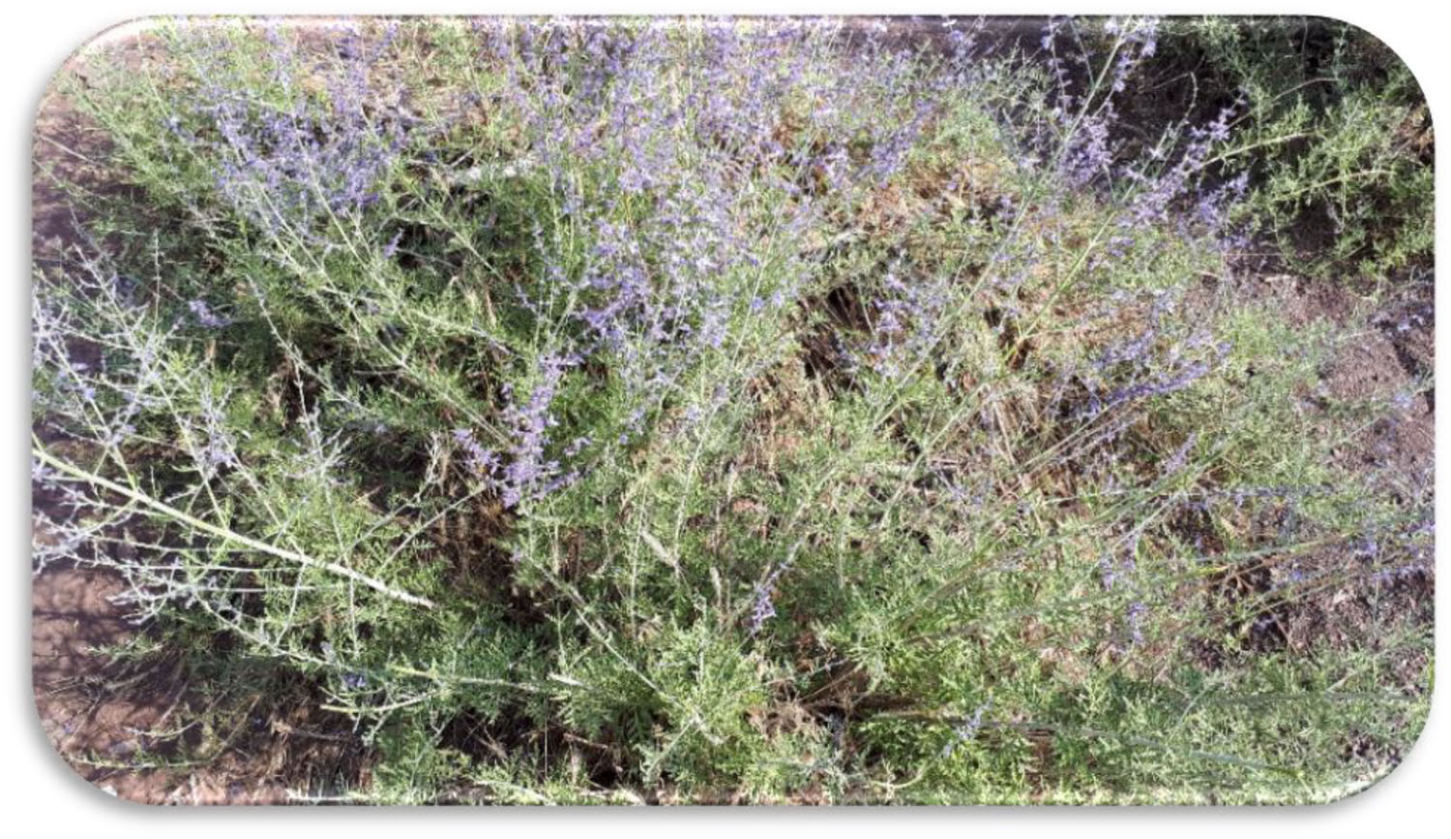
Whole plant of *Perovskia abrotanoides* Kar. at the flowering stage.

Aerial parts of *P. abrotanoides* Kar. (Figure 1) were collected at the flowering stage (late July 2023) from the mountainous regions of Arzaneh village, Bakharz County, Iran. The collection site was located at an altitude of 1550 m above sea level (34°57′27″ N, 60°10′05″ E), with clay‐loam soil. Plant samples were immediately transferred to the laboratory and shade‐dried at room temperature until a constant weight was reached. For essential oil extraction, a portion of the shade‐dried plant material was mechanically ground to pass through a 2‐mm sieve.

Essential oil extraction was performed using water distillation in a Clevenger‐type apparatus (Lenz Laborglasinstrumente GmbH, Wertheim, Germany) for 3 h. Approximately 100 g of air‐dried plant material was used for each distillation, and extraction was performed in triplicate to ensure reproducibility. The obtained essential oil was dried over anhydrous sodium sulphate to remove residual moisture, transferred into amber glass vials and stored at 4°C until analysis. Extraction yield was calculated as follows (Mahmodi et al. 2025):

$$\begin{array}{ccc} & & \mathrm{Essential}\mathrm{oil}\mathrm{yield}\left(\%\right)=\\ & & \left(\mathrm{Weight}\mathrm{of}\mathrm{essential}\mathrm{oil}\left(g\right)/\mathrm{Dry}\mathrm{weight}\mathrm{of}\mathrm{plant}\left(g\right)\right)\times 100 \end{array}$$

### 2.2 Chemical Characterization of the Essential Oil by GC–Flame Ionization Detector (FID) and GC/MS

Chemical composition of the essential oil was determined using an Agilent 7890A gas chromatograph (Agilent Technologies, Santa Clara, CA, USA) equipped with a FID and an Agilent 5975C mass selective detector (MSD). Separation was carried out on an HP‐5MS capillary column (30 m × 0.25 mm i.d., 0.25 µm film thickness; Agilent Technologies, Santa Clara, CA, USA). The oven temperature programme was as follows: initial 60°C for 2 min, then increased to 210°C at 3°C/min and finally to 240°C at 20°C/min, held for 8.5 min. Injector temperature was 240°C and detector temperature was 290°C for GC analysis. For GC/MS analysis, injector temperature was 280°C, ionization energy was 70 eV, and helium was used as carrier gas at a flow rate of 1 mL/min.

Identification of compounds was based on comparison of mass spectra with NIST and Wiley libraries, calculation of retention indices relative to *n*‐alkanes (C8–C24) and matching with reference data. Quantitative analysis was performed by area normalization without correction factors. Each essential oil sample was analysed in triplicate, and the average chromatographic peak areas were used for compound quantification.

### 2.3 Basal Diet and Rumen Fluid Collection

A basal diet (Table 1) containing 30% forage (alfalfa and clover) and 70% concentrate (barley, corn, soybean meal, sunflower meal, rice bran and sugar beet pulp) was formulated according to NRC (2007) recommendations for fattening sheep (30–40 kg body weight). This diet was not fed to the animals; rather, it was used as the substrate for in vitro incubations. The chemical composition (dry matter [DM] basis) was 16.6% crude protein, 2.28% ether extract, 4.53% ash, 48% non‐fibre carbohydrates, 1.1% Ca, 0.40% P and 2.35 Mcal/kg ME (Table 1).

**TABLE 1:** Ingredients and chemical analysis of the basal diet used in the rumen fermentation.

Ingredients	% of DM
Dried alfalfa	20
Clover forage	10
Barley grain	25
Corn grain	15
Sunflower meal	5
Sugar beet pulp	5
Rice bran	5
Soybean meal	10
Vitamin–mineral supplement^a^	2
Salt	1
Limestone	2
**Chemical composition (% of DM)**	
Dry matter (% of fresh weight)	81.2
Crude protein	16.6
Ether extract	2.28
Ash	4.53
Non‐fibre carbohydrates	48
Calcium	1.1
Phosphorus	0.4
Metabolizable energy (Mcal/kg DM)	2.35

Abbreviation: DM, dry matter.

^a^ Contains 8000 mg vitamin E, 100,000 IU vitamin A, 10,000 IU vitamin D3, 0.8 g 10% monensin, 0.4 g antioxidant, 74 g calcium, 15.6 g sodium, 8 mg cobalt, 0.15 g copper, 1 g manganese, 6 mg selenium, 30 mg iodine, 2 g zinc and 1 g iron.

Prior to chemical analyses, feed samples were ground using a laboratory cyclone mill (Cyclotec 1093, Foss Tecator, Hillerød, Denmark) fitted with a 1‐mm sieve. The chemical composition of the basal diet components was determined according to the standard protocols of AOAC (2005). DM was determined by drying samples at 105°C for 2 h (Method 930.15). Crude protein (CP; *N* × 6.25) was determined using the micro‐Kjeldahl method (Method 984.13; Kjeltec 2300, Foss Tecator, Hillerød, Denmark). Ether extract (EE) was measured using an automated Soxhlet extraction apparatus (Soxtherm, C. Gerhardt GmbH & Co. KG, Königswinter, Germany) with petroleum ether (Method 920.39). Ash content was determined by incineration in a muffle furnace (Nabertherm GmbH, Lilienthal, Germany) at 600°C for 2 h (Method 942.05). Neutral detergent fibre (NDF) was analysed according to Van Soest et al. (1991) using heat‐stable alpha‐amylase and sodium sulphite and expressed inclusive of residual ash.

Rumen fluid was obtained before the morning feeding from three male Baluchi sheep (body weight: 30 ± 3.5 kg) surgically fitted with permanent rumen cannulas. Rumen contents were sampled from multiple sites (cranial, central and caudal regions) within the rumen using a flexible stomach tube connected to a manual vacuum suction pump. The collected contents were immediately filtered through four layers of cheesecloth into a pre‐warmed (39°C) insulated thermos flask that had been pre‐flushed with continuous CO_2_ to maintain strict anaerobic conditions during transfer to the laboratory. Donor animals were fed a maintenance diet consisting of wheat straw (200 g/d) and a commercial concentrate (300 g/d; 50% ground barley, 25% wheat bran, 20% soybean meal, 3% limestone, 1% vitamin–mineral premix, 1% salt) providing 10.15 MJ/kg DM.

### 2.4 In Vitro Gas Production and Nutrient Degradability

The experiment was conducted as a completely randomized design consisting of six treatments corresponding to six levels of *P. abrotanoides* essential oil (0, 100, 200, 300, 400 and 500 mg/L culture medium). Each treatment was represented by four incubation bottles (experimental units) per run, and the entire experiment was repeated in two independent incubation runs performed on different days using freshly collected rumen fluid. Therefore, a total of 48 incubation bottles (6 treatments × 4 replicates × 2 runs) were included in the experiment. Mean values obtained from the two incubation runs were used for statistical analysis to ensure analytical precision and reproducibility.

The artificial saliva (buffer solution) was prepared according to the standard protocol described by McDougall (1948). The buffer solution was maintained at 39°C and continuously saturated with CO_2_ until the pH stabilized at 6.8–6.9. Strained rumen fluid was then mixed with the artificial saliva buffer in a 1:2 (V/V) ratio under anaerobic conditions to prepare the final incubation medium. A 30 mL aliquot of this incubation medium along with 200 mg of the basal substrate (ground to pass through a 1‐mm sieve) was added to each bottle.

The bottles were sealed with rubber stoppers and aluminium caps and incubated at 39°C for 96 h. Incubation bottles were randomly assigned to incubation positions to minimize possible positional effects during incubation. Gas pressure and volume were recorded at 3, 6, 9, 12, 24, 48, 72 and 96 h according to the method of Theodorou et al. (1994) using a digital pressure transducer (Model PSA‐01‐RC1/8, Autonics, Busan, South Korea; measurement range: 0–100 kPa, accuracy: ±1% of full scale). Gas production data were fitted to the exponential model (Kazemi and Valizadeh 2023):

$$Y=b\left(1-e^{-ct}\right)$$

where *Y* is the gas produced at time *t* (mL), *b* is the asymptotic gas production potential (mL), *c* is the fractional rate of gas production (h^−1^), and *t* is the incubation time (h).

### 2.5 Post‐Incubation Sampling and Chemical Analyses

After 24 h of incubation, the pH was measured immediately using an electronic pH meter (Model HI2210‐01, Hanna Instruments, Woonsocket, RI, USA). Fermentation was stopped by placing the bottles on ice. The contents of each bottle were filtered through pre‐weighed polyester bags (pore size 45 µm) to separate the liquid and solid residues (Kazemi 2024).

The solid residue remaining in the bags was washed thoroughly with cold distilled water until the effluent ran clear and then dried at 60°C for 48 h to determine DM degradability. Subsequently, the dried residue was incinerated at 600°C for 2 h in a muffle furnace to determine organic matter degradability. The NDF degradability was evaluated by subjecting the un‐degraded solid residue to NDF analysis according to Van Soest et al. (1991) using heat‐stable α‐amylase.

For fluid analysis, total VFA concentration was determined by steam distillation using a Markham apparatus (Markham 1942). Methane volume was measured at 24 h by injecting 4 mL of 10 M NaOH into the headspace gas to absorb CO_2_, with the remaining gas considered as methane (Fievez et al. 2005). Ammonia nitrogen (NH_3_–N) concentration was determined by the micro‐Kjeldahl distillation method after mixing 5 mL of filtered culture fluid with 5 mL of 0.2 N HCl (AOAC 2005; Kazemi 2024). All analytical determinations were performed in duplicate.

### 2.6 Microbial Efficiency Indices

The partitioning factor (PF) and microbial mass yield (MMY) were calculated as (Kazemi and Saleh 2026)

- PF (mg truly digested OM/mL gas) = [Omi − (RDM − AshR)]/IVGP
- MMY (mg) = [OMi − (RDM − AshR)] − (IVGP × 2.2)

where Omi is the initial organic matter (mg), RDM is the residual dry matter (mg), AshR is the ash in residual matter (mg), and IVGP is the net gas production at 24 h (mL).

$$\mathrm{Microbial}\mathrm{synthesis}\mathrm{efficiency}\left(\%\right)=\left(\mathrm{MMY}/\mathrm{truly}\mathrm{digested}\mathrm{OM}\right)\times 100.$$

Total protozoa counts were determined using a Neubauer haemocytometer (Paul Marienfeld GmbH & Co. KG, Lauda‐Königshofen, Germany) after fixing 5 mL of culture fluid with 5 mL of 50% formalin and staining with brilliant green (Dehority 2003).

### 2.7 Statistical Analysis

Data were analysed using the GLM procedure of SAS (Version 9.4; SAS Institute Inc., Cary, NC, USA) according to the following statistical model:

$$\;Y_{ij}=\mu +T_{i}+\varepsilon_{ij}$$

where $Y_{ij}$ is the observed response, μ is the overall mean, $T_{i}$ is the fixed effect of essential oil level, and $\varepsilon_{ij}$ is the residual error.

Prior to analysis, the normality of residuals was evaluated using the Shapiro–Wilk test, and homogeneity of variances was assessed using Levene's test. When treatment effects were significant, means were compared using Tukey's multiple comparison test. In addition, orthogonal polynomial contrasts were performed to evaluate linear and quadratic responses to increasing essential oil supplementation levels. Statistical significance was declared at *p* < 0.05.

## 3 Results

### 3.1 Chemical Composition of Essential Oil

A total of 33 compounds were identified in the essential oil of *P. abrotanoides*, representing 99.4% of the total oil composition (Table 2). The essential oil yield was 1.5% (w/w) based on dry plant weight. The oil was dominated by oxygenated monoterpenes, with 1,8‐cineole (16.10%) and camphor (13.80%) accounting for nearly 30% of the total essential oil composition, followed by α‐pinene (8.40%), δ‐3‐carene (6.81%), *n*‐pentadecane (5.51%), (*E*)‐caryophyllene (4.21%) and α‐humulene (4.12%). The remaining constituents were present at concentrations below 4%.

**TABLE 2:** Chemical constituents identified in the essential oil of *Perovskia abrotanoides*.

No.	Chemical constituent	Amount (% W/W)	Retention index (RI)
1	α‐Thujene	0.40	924
2	α‐Pinene	8.4	932
3	Camphene	3.11	946
4	Sabinene	0.80	969
5	β‐Pinene	2.50	974
6	Myrcene	2.21	990
7	δ‐3‐Carene	6.81	1009
8	α‐Terpinene	1.30	1016
9	*p*‐Cymene	1.19	1024
10	1,8‐Cineole	16.1	1031
11	α‐Terpinolene	2.12	1085
12	Linalool	2.53	1095
13	Camphor	13.8	1141
14	α‐Terpineol	2.81	1188
15	Trans‐carveol	2.52	1217
16	*Cis*‐carveol	0.40	1229
17	Linalool acetate	0.11	1257
18	Bornyl acetate	2.83	1285
19	Carvacrol	0.12	1300
20	Methyl decanoate	1.12	1321
21	α‐Cubebene	1.31	1371
22	α‐Gurjunene	0.34	1410
23	(*E*)‐Caryophyllene	4.21	1421
24	α‐Humulene	4.12	1455
25	α‐Amorphene	2.13	1481
26	*N*‐pentadecane	5.51	1500
27	β‐Bisabolene	0.21	1507
28	γ‐Cadinene	0.71	1514
29	δ‐Cadinene	2.51	1524
30	Caryophyllene oxide	0.45	1582
31	β‐Cedrene epoxide	1.51	1611
32	Epi‐α‐cadinol	2.23	1642
33	(*E*,*Z*)‐farnesol	2.01	1742

### 3.2 Gas Production Parameters and Methane Yield

Increasing levels of *P. abrotanoides* essential oil significantly reduced methane production by 57.5%, decreasing from 15.00 mL in the control treatment to 6.37 mL at 500 mg/L (linear effect, *p* < 0.0001) (Table 3). Likewise, gas production potential declined by 44.7%, whereas cumulative gas production after 24, 48, 72 and 96 h decreased by 47.9%, 47.0%, 43.7% and 44.2%, respectively, with increasing essential oil concentration (all linear effects, *p* < 0.0001). The fractional rate of gas production was also affected by treatment, with the lowest value observed at 500 mg/L (0.0848 h^−1^), corresponding to a 12.5% reduction compared with the control (cubic and quartic effects, *p* = 0.03). Significant quadratic responses were observed for gas production potential and cumulative gas production parameters (*p* = 0.0012–0.0030), whereas quartic effects were not significant.

**TABLE 3:** Effect of different levels of *Perovskia abrotanoides* essential oil on gas production parameters and methane yield.

Essential oil level (mg/L)	Methane^1^ (mL)	Fractional rate of gas production (h^−1^)	Gas production potential (mL)	24‐h gas production (mL)	48‐h gas production (mL)	72‐h gas production (mL)	96‐h gas production (mL)
0 (control)	15.00^a^	0.0969^a^	72.23^a^	59.32^a^	69.80^a^	74.17^a^	74.95^a^
100	13.22^b^	0.0920^b^	61.34^b^	49.83^b^	59.18^b^	62.95^b^	63.33^b^
200	11.35^c^	0.0993^a^	56.08^c^	46.00^c^	54.22^c^	57.87^c^	58.32^c^
300	9.70^d^	0.0978^a^	50.08^d^	40.35^d^	48.27^d^	51.97^d^	52.22^d^
400	7.57^e^	0.0965^ab^	44.29^e^	35.97^e^	42.47^e^	45.75^e^	46.27^e^
500	6.37^e^	0.0848^c^	39.92^f^	30.89^f^	36.99^f^	41.74^f^	41.83^f^
SEM	0.44	0.0012	2.26	0.66	0.64	0.69	0.70
***p* value**							
Linear	<0.0001	0.34	<0.0001	<0.0001	<0.0001	<0.0001	<0.0001
Quadratic	0.77	0.81	0.0012	0.003	0.002	0.0023	0.0010
Cubic	0.79	0.03	0.0176	0.05	0.01	0.0084	0.0090
Quartic	0.78	0.03	0.20	0.07	0.16	0.21	0.14

*Note*: Different superscripts (^a–f^) within each column indicate significant differences between means at *p* < 0.05.

Abbreviation: SEM, standard error of the mean.

^1^ Methane production was measured after 24 h of incubation.

### 3.3 Protozoa Population and Fermentation Parameters

Increasing supplementation levels of *P. abrotanoides* essential oil resulted in a 33.8% reduction in total protozoal population, decreasing from 5.17 × 10^5^ to 3.42 × 10^5^ cells/mL (linear effect, *p* < 0.0001) (Table 4). Total VFA concentration decreased by 17.4%, whereas NH_3_–N concentration decreased by 29.4% relative to the control treatment (linear effect, *p* < 0.0001). In contrast, ruminal pH increased from 6.63 to 6.85, representing an increase of approximately 3.3%. No significant quadratic, cubic or quartic responses were detected for these variables (*p* > 0.05).

**TABLE 4:** Effect of different levels of *Perovskia abrotanoides* essential oil on protozoa population and fermentation parameters after 24 h of incubation.

Essential oil level (mg/L)	Total protozoa (×10^5^ cells/mL)	Total volatile fatty acid concentration (mmol/L)	NH_3_–N concentration (mg/dL)	pH
0 (control)	5.17^a^	96.00^a^	24.25^a^	6.63^d^
100	4.95^a^	92.75^a^	23.27^a^	6.65^cd^
200	4.52^b^	89.12^b^	21.87^b^	6.69^c^
300	4.22^b^	85.62^c^	19.75^c^	6.77^b^
400	3.87^c^	82.25^cd^	18.62^c^	6.80^b^
500	3.42^d^	79.25^d^	17.12^d^	6.85^a^
SEM	0.13	1.28	0.55	0.02
***p* value**				
Linear	<0.0001	<0.0001	<0.0001	<0.0001
Quadratic	0.76	0.98	0.54	0.34
Cubic	0.66	0.89	0.31	0.28
Quartic	0.58	0.96	0.58	0.39

*Note*: Different superscripts (^a–d^) within each column indicate significant differences between means at *p* < 0.05.

Abbreviation: SEM, standard error of the mean.

### 3.4 Nutrient Degradability and Microbial Efficiency Indices

Supplementation with *P. abrotanoides* essential oil markedly improved microbial efficiency indices (Table 5). Microbial biomass production increased by 45.1%, microbial synthesis efficiency increased by 79.6% and PF increased by 55.3% compared with the control treatment (all linear effects, *p* < 0.0001). Conversely, NDF degradability, OM degradability and DM degradability decreased by 19.4%, 14.0% and 11.9%, respectively, as essential oil concentration increased. A significant quadratic effect was observed only for DM degradability (*p* = 0.025), whereas no other quadratic, cubic or quartic effects were detected (*p* > 0.05).

**TABLE 5:** Effect of different levels of *Perovskia abrotanoides* essential oil on nutrient degradability indices and microbial efficiency parameters.

Essential oil level (mg/L)	Microbial biomass production (mg)	Microbial biomass synthesis efficiency (%)	Partitioning factor (mg truly degraded organic matter/mL gas produced)	Neutral detergent fibre degradability (%)	Organic matter degradability (%)	Dry matter degradability (%)
0 (control)	58.32^d^	30.87^f^	3.18^e^	50.25^a^	75.01^a^	73.75^a^
100	63.46^c^	36.67^e^	3.47^d^	48.50^a^	73.01^ab^	71.25^b^
200	66.88^c^	39.80^d^	3.65^d^	46.50^b^	70.50^b^	67.50^c^
300	75.06^b^	45.81^c^	4.06^c^	44.75^b^	67.50^c^	67.25^cd^
400	76.44^b^	49.09^b^	4.33^b^	42.50^c^	67.25^c^	66.50^cd^
500	84.63^a^	55.46^a^	4.94^a^	40.50^d^	64.50^c^	65.00^d^
SEM	1.93	1.72	0.12	0.74	0.83	0.68
***p* value**						
Linear	<0.0001	<0.0001	<0.0001	<0.0001	<0.0001	<0.0001
Quadratic	0.65	0.48	0.47	0.76	0.42	0.025
Cubic	0.34	0.98	0.91	0.91	0.31	0.76
Quartic	0.20	0.11	0.24	0.82	0.70	0.19

*Note*: Different superscripts (^a–f^) within each column indicate significant differences between means at *p* < 0.05.

Abbreviation: SEM, standard error of the mean.

## 4 Discussion

The present study investigated the chemical composition of *P. abrotanoides* essential oil and its effects on in vitro rumen fermentation parameters, methane production, protozoa population, nutrient degradability and microbial efficiency indices. The results demonstrated that the essential oil, rich in oxygenated monoterpenes (mainly 1,8‐cineole and camphor), significantly reduced methane production and protozoa population while increasing microbial biomass synthesis efficiency and PF, albeit with some adverse effects on fibre degradability at higher doses. These findings support the hypothesis that *P. abrotanoides* essential oil, through its bioactive antimicrobial constituents, can establish a balance between methane reduction and maintenance of rumen fermentation efficiency at an appropriate dosage level.

### 4.1 Chemical Composition of *Perovskia abrotanoides* Essential Oil

The GC–MS analysis of the essential oil (Table 2) revealed 33 compounds, with 1,8‐cineole (16.10%), camphor (13.80%), α‐pinene (8.40%), δ‐3‐carene (6.81%) and (*E*)‐caryophyllene (4.21%) as the major constituents. The essential oil yield was 1.5% (w/w) based on dry plant weight. This yield is comparable to previously reported values for *P. abrotanoides* from different regions of Iran. Shahraki et al. (2013) reported essential oil yields of 1.8% and 1.6% for samples collected from Kiasar (Mazandaran) and Golestan National Park, respectively. Similarly, Pourhosseini et al. (2018) observed yields ranging from 0.7% in stalks to 2.3% in flowers, with leaf yield of 1.0%. Ghaffari et al. (2018) analysed 17 populations across Iran and reported a broader yield range of 1.25%–3.61%, with the highest yield (3.61%) recorded in a Semnan population and the lowest (1.25%) in an Isfahan population (Keshe). The yield obtained in the present study (1.5%) falls well within these reported ranges, confirming that *P. abrotanoides* essential oil yield is influenced by geographical origin, climatic conditions and plant part analysed, while remaining consistently moderate across different regions.

These findings are consistent with previous reports on *P. abrotanoides* essential oil composition from various regions of Iran. Morteza‐Semnani (2004) reported camphor (34.1%), 1,8‐cineole (18%) and α‐pinene (6.5%) as the main components. Similarly, Mahboubi and Kazempour (2009) identified camphor (23%), 1,8‐cineole (22%) and α‐pinene (12%) in their sample from Kashan, Iran. Ghaffari et al. (2018) analysed 17 populations of *P. abrotanoides* from different Iranian provinces and found camphor (4.05%–35.94%), 1,8‐cineole (7.15%–24.34%), borneol (0%–21.75%) and α‐pinene (2.05%–10.33%) as the main constituents. Our results fall within these reported ranges, confirming that *P. abrotanoides* essential oil is characterized by high levels of oxygenated monoterpenes, particularly 1,8‐cineole and camphor.

However, considerable variation exists in the chemical composition of *P. abrotanoides* essential oil depending on geographical origin, climatic conditions, altitude, soil properties and plant part analysed. Pourhosseini et al. (2018) reported that flower essential oil contained higher amounts of camphor (18.8%) and 1,8‐cineole (16.5%) compared to leaves (10.1% and 11.4%, respectively) and stalks (21.0% and 16.3%, respectively). Shahraki et al. (2013) also found significant differences between two natural habitats in Golestan and Mazandaran provinces, with the Kiasar region (Mazandaran) showing higher essential oil yield (1.8%) and a different major compound profile (higher 1,8‐cineole and camphor) compared to Golestan National Park (1.6%). In contrast, one study from the same Kiasar region reported α‐terpineol (21.9%–32.0%) and *n*‐octanol (17.4%–23.3%) as the dominant compounds (Kolbady Nejad et al. 2013), indicating the existence of different chemotypes within the species. Ashraf et al. (2014) reported an entirely different profile from Pakistan, with (*E*)‐9‐dodecenal (66.5%) as the major component in stem essential oil, highlighting the profound impact of geographical and genetic factors on essential oil composition. These variations are attributable to differences in genotype, agronomic practices, climatological factors, developmental stage, post‐harvest storage and extraction methods (Ashraf et al. 2014).

### 4.2 Effect on Methane Production and Gas Production Parameters

Increasing levels of *P. abrotanoides* essential oil reduced methane production by approximately 57.5%. This reduction is most likely attributable to the high concentration of oxygenated monoterpenes, particularly 1,8‐cineole and camphor, which are known to suppress methanogenic archaea directly and indirectly through inhibition of ruminal protozoa. The lipophilic nature of these compounds disrupts microbial cell membranes and alters hydrogen metabolism, thereby limiting the substrate available for methanogenesis. These findings are consistent with previous studies demonstrating the antimethanogenic potential of essential oils rich in oxygenated monoterpenes (Cobellis et al. 2016; Nunes et al. 2023; Kelly and Kebreab 2023).

Patra and Yu (2012) evaluated five essential oils (clove, eucalyptus, garlic, *origanum* and peppermint) at doses up to 1.0 g/L and reported methane reductions ranging from 17.6% (eucalyptus) to 87% (origanum). Although our *P. abrotanoides* essential oil contains 1,8‐cineole (a major component of eucalyptus oil), its methane reduction efficacy (57.5% at 500 mg/L) was considerably higher than that reported for pure eucalyptus oil (17.6% at 1.0 g/L) by Patra and Yu (2012). This enhanced efficacy may be attributed to the synergistic effects of multiple bioactive compounds in *P. abrotanoides* essential oil, including camphor, α‐pinene and δ‐3‐carene, in addition to 1,8‐cineole.

Benetel et al. (2022) tested 10 essential oils in an in vitro rumen fermentation system and found that oregano and white thyme essential oils caused drastic reductions in total gas production (up to 75%) and methane production, with net methane values dropping from 6.92 mL (control) to 0.17 mL (oregano) and 0.57 mL (white thyme) at 500 mg/L. These reductions were attributed to the high content of thymol and carvacrol, phenolic monoterpenes with strong antimicrobial activity. Although our essential oil does not contain thymol or carvacrol, its high content of 1,8‐cineole and camphor appears to confer substantial antimethanogenic activity, albeit less potent than oregano oil.

The mechanism by which essential oils reduce methane production is primarily through direct inhibition of methanogenic archaea and/or indirect suppression of protozoa, which are symbiotically associated with methanogens (Patra and Yu 2012). In the present study, total protozoa population decreased linearly (*p* < 0.0001) from 5.17 × 10^5^ cells/mL in the control to 3.42 × 10^5^ cells/mL at 500 mg/L (Table 4). This antiprotozoal effect likely contributed to the observed reduction in methane production, as protozoa provide hydrogen for methanogens. Patra and Yu (2012) similarly reported that all tested essential oils reduced protozoal abundance, with origanum and peppermint oils causing the greatest reductions (nearly 3 log units). The antiprotozoal activity of essential oils is attributed to their lipophilic nature, which allows them to penetrate the protozoal cell membrane and disrupt cellular functions (Patra and Yu 2012).

### 4.3 Effect on Protozoa Population and Fermentation Parameters

Total protozoa population decreased linearly (*p* < 0.0001) with increasing essential oil levels, from 5.17 × 10^5^ cells/mL in the control to 3.42 × 10^5^ cells/mL at 500 mg/L (Table 4). This marked reduction is likely attributable to the antimicrobial activity of the major oxygenated monoterpenes present in the essential oil, particularly 1,8‐cineole and camphor. Owing to their lipophilic nature, these compounds can penetrate protozoal cell membranes, disrupt membrane integrity, increase permeability and interfere with essential metabolic processes, ultimately reducing protozoal survival and activity in the rumen ecosystem. This finding is consistent with Mahboubi and Kazempour (2009), who demonstrated that *P. abrotanoides* essential oil and its principal constituents (camphor and α‐pinene) possess strong antimicrobial activity. Although protozoa were not evaluated in their study, the proposed mechanism of membrane disruption agrees with previous reports describing the mode of action of essential oils against rumen microorganisms (Patra and Yu 2012). The reduction in protozoal abundance may also partially explain the lower methane production observed in the present study because ruminal protozoa maintain a close symbiotic association with methanogenic archaea and contribute substantially to hydrogen transfer required for methanogenesis.

Total VFA concentration decreased linearly (*p* < 0.0001) from 96.00 mmol/L in the control to 79.25 mmol/L at 500 mg/L (Table 4). This decrease most likely reflects a reduction in overall microbial fermentation activity resulting from partial inhibition of fermentative microorganisms, particularly cellulolytic bacteria responsible for carbohydrate degradation. Lower fermentation activity inevitably reduces the production of fermentation end‐products, including VFA. This observation agrees with Patra and Yu (2012), who reported decreased total VFA concentrations following supplementation with clove and oregano essential oils, whereas garlic, eucalyptus and peppermint oils produced little or no effect. Although reduced VFA production may indicate some depression of fermentative activity at higher doses, it also suggests that the antimethanogenic effect of the essential oil is accompanied by a shift in rumen fermentation pathways rather than complete inhibition of microbial metabolism.

The NH_3_–N concentration decreased linearly (*p* < 0.0001) from 24.25 mg/dL in the control to 17.12 mg/dL at 500 mg/L (Table 4). The reduction in ammonia concentration is likely associated with inhibition of hyper‐ammonia‐producing bacteria, thereby reducing amino acid deamination and improving nitrogen retention within the microbial ecosystem. Such an effect may increase the availability of nitrogen for microbial protein synthesis instead of ammonia accumulation. Patra and Yu (2012) similarly reported lower ammonia concentrations after supplementation with clove and oregano essential oils, attributing this response to reduced deamination activity. Mahboubi and Kazempour (2009) also demonstrated the antimicrobial activity of camphor and α‐pinene, suggesting that these compounds may inhibit ammonia‐producing microorganisms. Importantly, NH_3_–N concentrations remained above the minimum threshold required for optimal microbial growth (Satter and Slyter 1974), indicating that nitrogen availability for microbial protein synthesis was not compromised despite the reduction in ammonia concentration.

In contrast, pH increased linearly (*p* < 0.0001) from 6.63 in the control to 6.85 at 500 mg/L (Table 4). The increase in ruminal pH is a logical consequence of reduced fermentation intensity and lower VFA production, as fewer organic acids accumulated in the incubation medium. Maintaining ruminal pH within this range may also contribute to a more stable rumen environment by reducing the risk of excessive acidification. Similar increases in ruminal pH following essential oil supplementation have been reported by Patra and Yu (2012). Therefore, the observed increase in pH appears to represent an indirect response to changes in fermentation activity rather than a direct effect of the essential oil itself.

### 4.4 Effect on Nutrient Degradability and Microbial Efficiency

Increasing levels of essential oil linearly decreased (*p* < 0.0001) NDF degradability (from 50.25% to 40.50%), OM degradability (from 75.01% to 64.50%) and DM degradability (from 73.75% to 65.00%) (Table 5). The reduction in nutrient degradability is likely associated with inhibition of fibrolytic microorganisms responsible for structural carbohydrate degradation. Oxygenated monoterpenes, such as 1,8‐cineole and camphor, may suppress the growth and enzymatic activity of cellulolytic bacteria, thereby limiting fibre digestion and reducing substrate degradation. This inhibitory effect becomes more pronounced at higher essential oil concentrations because antimicrobial activity extends beyond methanogens to beneficial fibrolytic microorganisms. These findings agree with Patra and Yu (2012), who reported reductions in apparent DM and NDF degradability following supplementation with several essential oils. Likewise, Benetel et al. (2022) observed decreased in vitro DM digestibility with white thyme essential oil. Therefore, although the essential oil effectively mitigated methane production, excessive supplementation may compromise nutrient utilization, highlighting the importance of identifying an optimal inclusion level that balances environmental benefits with digestive efficiency.

Despite the reduction in fibre degradability, microbial biomass production increased linearly (*p* < 0.0001) from 58.32 mg in the control to 84.63 mg at 500 mg/L (Table 5). Similarly, microbial biomass synthesis efficiency increased from 30.87% to 55.46%, and the PF increased from 3.18 to 4.94 mg truly degraded OM/mL gas produced. These responses indicate that microbial metabolism became more efficient, allowing a greater proportion of degraded substrate to be incorporated into microbial biomass rather than being lost as gaseous fermentation products. Inhibition of methanogenesis reduces energy losses and may redirect reducing equivalents towards alternative metabolic pathways and microbial growth. Consequently, the remaining microbial community may utilize available nutrients more efficiently despite lower overall substrate degradation. Similar improvements in PF following essential oil supplementation have been reported by Benetel et al. (2022), who suggested that increased PF reflects enhanced microbial efficiency. Patra and Yu (2012) also proposed that inhibition of methanogenesis may alter hydrogen utilization pathways and promote the growth of alternative microbial groups. Collectively, these findings suggest that *P. abrotanoides* essential oil has the potential to improve microbial energy utilization efficiency, although this beneficial effect should be balanced against the observed reduction in fibre degradability when determining the optimal supplementation level.

## 5 Conclusion

The present study demonstrated that *P. abrotanoides* essential oil, characterized by a high content of oxygenated monoterpenes, effectively modulated in vitro rumen fermentation by reducing methane production and protozoal populations while improving microbial biomass production and microbial synthesis efficiency. However, higher supplementation levels also reduced nutrient degradability, indicating that excessive doses may adversely affect rumen fermentation. Among the tested concentrations, 200–300 mg/L appeared to provide the most favourable balance between methane mitigation and fermentation efficiency under the experimental conditions.

A major limitation of this study is that the findings were obtained under in vitro conditions, which may not fully represent the complexity of the in vivo rumen environment. Therefore, further in vivo studies are required to confirm the efficacy, safety, optimal supplementation level and long‐term effects of *P. abrotanoides* essential oil on animal performance, nutrient utilization and enteric methane emissions before practical application in ruminant production systems can be recommended.

## Author Contributions

Mohsen Kazemi was solely responsible for the conceptualization, data curation, literature search, synthesis of information, drafting of the manuscript and final editing.

## Funding

The author has nothing to report.

## Ethics Statement

Rumen fluid was collected from three rumen‐fistulated sheep in accordance with institutional guidelines for the care and use of animals. The experimental procedures were reviewed and approved by the Animal Ethics Committee in University of Torbat‐e Jam, Approval no. 19293.

## Conflicts of Interest

The author declares no conflicts of interest.

## Data Availability

All relevant data supporting the findings of this study are included within the manuscript, and additional raw datasets are available from the corresponding author upon reasonable request.
